# A novel cap-assisted electrohydraulic lithotripsy technique for common bile duct stones impacted in the duodenum

**DOI:** 10.1055/a-2873-9071

**Published:** 2026-06-01

**Authors:** Tomohiro Ishii, Kazuya Sugimori, Arisa Omata, Hideyuki Anan, Takashi Kurosawa, Shin Maeda

**Affiliations:** 1 Department of Gastroenterology 89460 Saiseikai Yokohamashi Nanbu HospitalYokohamaJapan; 2 Department of GastroenterologyYokohama City University Graduate School of MedicineYokohamaJapan

Surgery or electrohydraulic lithotripsy (EHL) is a treatment option for large stones impacted in the duodenum 1 2 . However, large stones may require multiple sessions or take a long time 3 4 . We report the successful and efficient treatment of large common bile duct stones impacted in the duodenum using a novel cap-assisted EHL technique.

**Fig. 1:**
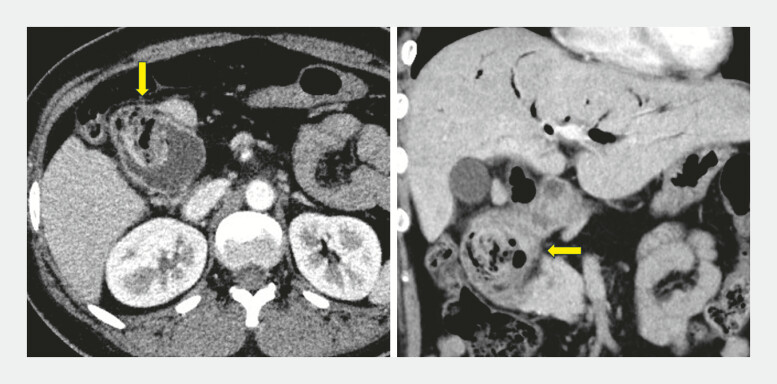
Arterial phase of contrast-enhanced computed tomography (CT) revealed that a large stone in the dilated bil duct partially protruded into the duodenum in the axial view (left). The coronal CT scan revealed pneumobilia in the intrahepatic bile ducts; a large stone partially protruded into the duodenum (right).

**Fig. 2:**
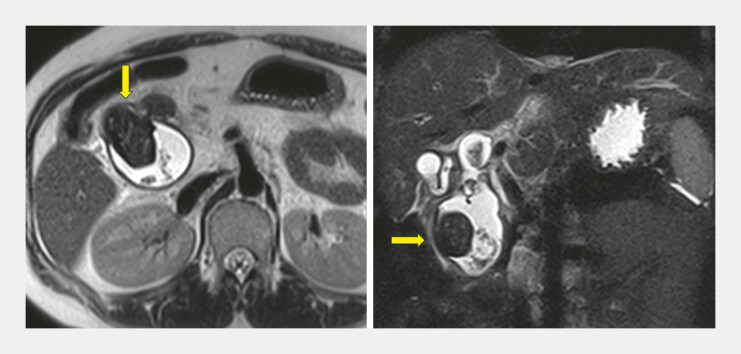
In an axial view, T2-weighted magnetic resonance imaging showed a large stone in the dilated common bile duct protruding into the duodenum (left). A coronal view revealed multiple stones in the dilated common bile duct (right).

**Fig. 3:**
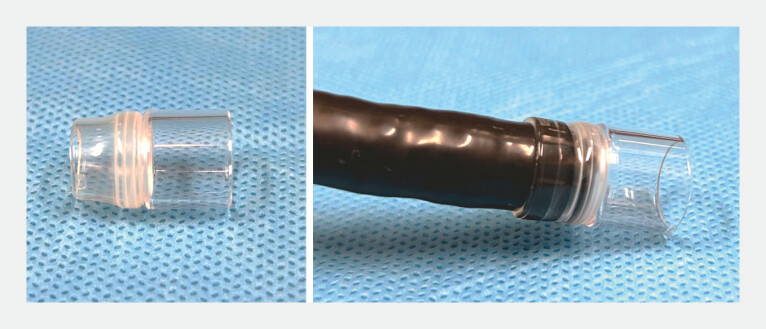
A long cylindrical cap (MH-463, Olympus, Tokyo, Japan; left) was attached to the forward-viewing endoscope (GIF-H290T; Olympus, Tokyo, Japan; right).

**Fig. 4:**
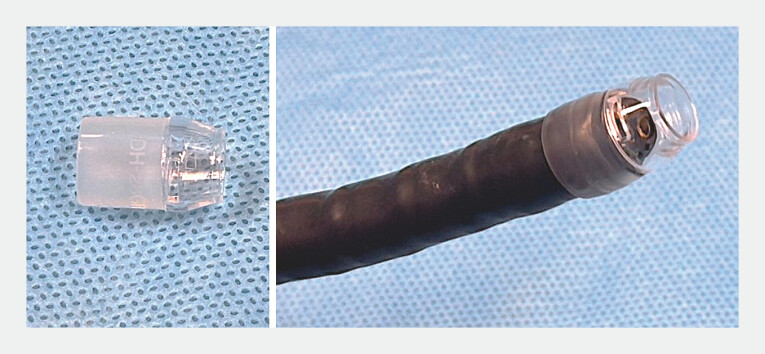
A short, small-caliber-tip transparent hood (DH-28GR, Fujifilm, Japan; left) was attached to the forward-viewing endoscope (GIF-H290T; Olympus, Tokyo, Japan; right).

A 31-year-old woman underwent choledochoduodenostomy during childhood for congenital biliary dilatation. She presented with epigastric pain and nausea and was admitted for cholangitis. Abdominal computed tomography and magnetic resonance cholangiopancreatography revealed a 4.5-cm stone protruding from the bile duct through the anastomosis into the duodenum ( Fig. 1 , Fig. 2 ). Endoscopic retrograde cholangiopancreatography (ERCP) was attempted; however, the scope could not be passed through because the stone was impacted in the descending duodenum and choledochoduodenal anastomosis could not be visualized. Since the bile flow was observed from the 6 oʼclock position of the stone, a catheter was inserted; successful bile duct intubation was achieved. The impacted stone was not dislodged; however, we placed biliary stents and treated cholangitis. After cholangitis improved, ERCP was performed. A long cylindrical cap (MH-463; Olympus, Tokyo, Japan; Fig. 3 ) was attached to a forward-viewing endoscope (GIF-H290T; Olympus, Tokyo, Japan). The cap was pressed against the stone; EHL was performed while slowly injecting saline into the cap through an auxiliary water channel. Saline leaked from the stone cracks, preventing its accumulation in the long cap. Therefore, we replaced the cap with a short, small-caliber-tip transparent hood (DH-28GR; Fujifilm, Japan; Fig. 4 ), filled the small space with saline, and continued the EHL ( Video 1 ). The stone was efficiently fragmented and successfully removed within 90 minutes. A few reports describe EHL with a forward-viewing endoscope while injecting saline into the cap. Using this technique, saline accumulates only within the cap, allowing safe procedures without aspiration, even in cases of gastrointestinal obstruction.

The successful and efficient treatment of large common bile duct stones impacted in the duodenum using a novel cap-assisted EHL technique.Video 1

Endoscopy_UCTN_Code_TTT_1AO_2AL
